# Mannose-Binding Lectin Deficiency Is Associated with Myocardial Infarction: The HUNT2 Study in Norway

**DOI:** 10.1371/journal.pone.0042113

**Published:** 2012-07-27

**Authors:** Inga Thorsen Vengen, Hans O. Madsen, Peter Garred, Carl Platou, Lars Vatten, Vibeke Videm

**Affiliations:** 1 Department of Laboratory Medicine, Children's and Women's Health, Norwegian University of Science and Technology. Trondheim, Norway; 2 Laboratory of Molecular Medicine, Department of Clinical Immunology, Faculty of Health Sciences, University of Copenhagen, Copenhagen, Denmark; 3 Department of Internal Medicine, Levanger Hospital, Nord-Trøndelag Health Trust, Levanger, Norway; 4 Department of Public Health, Norwegian University of Science and Technology, Trondheim, Norway; 5 Department of Immunology and Transfusion Medicine, Trondheim University Hospital, Trondheim, Norway; Albert Einstein College of Medicine, United States of America

## Abstract

**Objectives:**

Mannose-binding lectin (MBL) and ficolins activate the complement cascade, which is involved in atherogenesis. Based on a pilot study, we hypothesized that functional polymorphisms in the MBL gene (*MBL2*) leading to dysfunctional protein are related to development of myocardial infarction (MI). The aim of the present study was to study polymorphisms in *MBL2* and ficolin genes in relation to the risk of MI.

**Methods and Results:**

Using the population-based HUNT Study in Norway, 57133 persons were followed up for a first-time MI from 1995–1997 until the end of 2008. The 370 youngest MI patients were matched by age (range 29–62 years) and gender to 370 controls. A younger population was selected because disease in this group might be less dependent on non-genetic risk factors. The study size was based on power calculation. Polymorphisms in *MBL2* and in the genes of ficolin-1, ficolin-2 and ficolin-3 were genotyped by pyrosequencing and related to the risk of MI, estimated as odds ratios (OR). Functional haplotypes were analyzed and stringent alpha levels of significance were set by permutation testing. Variant *MBL2* haplotypes causing MBL deficiency were associated with a two-fold higher risk of MI (OR 2.04, 95%CI 1.29–3.24). Adjustments for conventional cardiovascular risk factors did not substantially influence the association. The ficolins were not associated with MI risk.

**Conclusion:**

In a young to middle aged and relatively healthy Caucasian population, *MBL2* variants related to functional MBL deficiency were associated with a doubling of the risk for MI, independent of conventional risk factors. This supports that MBL deficiency may lead to increased atherosclerosis or development of vulnerable plaques.

## Introduction

Accumulating evidence suggests that atherosclerosis is an inflammatory disease where the innate immune system plays a crucial part in the pathophysiology [1]. The complement system is involved at different stages of atherosclerosis, from the early formation of fatty streaks [2] until destabilization of mature plaques. In addition to enhancing the atherosclerotic process, activation of the complement cascade may also have a protective effect by removing cell debris and immune complexes from the atherosclerotic lesions.

The complement system is activated through three possible pathways, denoted as the classic, alternative or lectin pathway. The latter is initiated by mannose-binding lectin (MBL) or by proteins of the ficolin family [3]. During the last decade MBL has received attention as a potential marker of atherosclerosis. The MBL gene (*MBL2*) has several polymorphic sites [4], and the combined genetic profile corresponds to *normal*, *intermediate* or *deficient* serum concentrations of the protein [5]. After the original study by Madsen et al in 1998, where MBL deficiency was associated with increased risk for severe atherosclerosis in relatively young patients [6], polymorphisms in *MBL2* and serum concentrations of the protein have been linked to both increased and reduced risk of atherosclerosis and coronary artery disease in different populations [7], [8], [9], [10], [11]. Furthermore, a study in knockout mice demonstrated increased atherosclerotic lesions when the lectin pathway was inhibited [12].

In a pilot study including patients with stable angina pectoris, we found an increased frequency of variant *MBL2* haplotypes corresponding to MBL deficiency in patients with significant coronary artery stenosis compared to patients without significant stenosis, as detailed below. Based on the results from that study, we hypothesised that MBL deficiency is related to the development of myocardial infarction (MI) and that variations in the genes coding for MBL and ficolins may be associated with atherosclerosis.

We therefore performed a case-control study, assessing the association of *MBL2* and ficolin genotypes with the risk of a first-time MI at young and middle age, i.e. at an age when the genetic influence may be stronger and less dependent on non-genetic risk factors than at older age. The study showed that MBL deficiency was associated with a doubling of the risk for MI, independent of conventional risk factors. This supports a protective role of MBL in atherosclerosis.

## Methods

### Ethics statement

The study protocols conformed to the Helsinki declaration. Both studies were approved by the Regional Research Ethics Committee in Medicine, Central Norway (reference 157-1997 dated 06/11/1997 and reference 2009/1852-2, dated 11/20/2009), and the case-control study was approved by the Data Inspectorate of Norway. The participants in the pilot study gave written informed consent. The HUNT2 participants had signed consent to participate in morbidity and mortality follow-up studies. No minors or children participated in the present study.

The pilot study was based on data from 236 adults (146 males and 90 females) in a study on circulating plasma markers of inflammation, admitted for first-time elective coronary angiography due to suspected coronary artery disease as detailed previously [13]. Later, data on other genetic markers in this population have also been published [14], [15]. Briefly, the patients with available samples for the present pilot study (n = 234 due to 2 lost samples) consisted of 131 patients with significant (>50%) artery stenosis in at least one main coronary artery branch and 103 patients without significant coronary artery stenosis, as demonstrated by quantitative coronary angiography. Information was also registered about classical cardiovascular risk factors (body mass index (BMI), smoking, hypertension, hypercholesterolemia, and diabetes). DNA was extracted from blood samples anticoagulated with ethylenediaminetetraacetic acid by a salting-out procedure and genotyping for *MBL2* polymorphisms was performed as described in [16]. Plasma was kept at −70°C and later analyzed for MBL concentrations using an in-house enzyme-linked immunoassay [17].

The case-control study was generated by linkage of population data from the second wave of the Nord-Trøndelag Health Study (HUNT2) to validated information on incident acute MIs.

HUNT2 was carried out in 1995–1997 as a population-based study and information was collected through comprehensive questionnaires and a clinical examination. The county of Nord-Trøndelag is fairly representative for Norway as a whole, with only 3% non-Caucasians. All inhabitants 13 years of age and older were invited, and a venous blood sample was drawn from all persons 20 years of age and older. In total, about 75 000 (70%) of those invited attended the study. The inclusion process is described elsewhere [18].

There are two primary referral hospitals in the county of Nord-Trøndelag (Levanger Hospital and Namsos Hospital). Data on all acute MI hospitalizations from 1995 (corresponding to the commencement of HUNT2) to the end of 2000 were registered retrospectively, whereas from 2001 registration has been done prospectively. MI was diagnosed according to the European Society of Cardiology/American College of Cardiology consensus guidelines [19]. The criteria were elevated troponin T or troponin I at the same time course with at least one of the following criteria: 1) symptoms consistent with myocardial infarction and/or 2) ECG changes with development of significant Q wave and/or 3) ECG changes consistent with ischemia (ST-segment elevation or depression).

Among participants in HUNT2, the following criteria had to be met to be eligible for the present study: available DNA, and no previous self-reported MI, angina pectoris or stroke. In total, 57 133 individuals met these criteria. We linked these HUNT2 participants to the hospital registrations to ascertain incident cases of MI from baseline at HUNT2 until the end of 2008. During follow-up, 1689 individuals had experienced an MI. Among incident MI patients, the 370 youngest were selected as cases in the study. As controls, we randomly selected 370 participants who were matched to the cases by age (±2 years) and gender. All controls were at risk of MI at the time when the MI occurred in their respective matched case.

### Clinical information

Measurements of blood pressure, height, weight, waist and hip circumference were done as previously described [18]. BMI and waist-hip ratio (WHR) were calculated. Concentrations of blood lipids, creatinine and glucose were analysed by standard methods at the Central Laboratory at Levanger Hospital. Hypertension was defined as systolic blood pressure ≥140 mmHg or as diastolic blood pressure ≥90 mmHg, or as current use of antihypertensive medication. Information on use of other medications, such as statins or anti-platelet therapy was not available. Hypercholesterolemia was defined as total cholesterol >6.2 mmol/L. Smoking was classified in three groups: never, former or current smokers. A report of MI before 60 years of age in first-degree relatives was considered as a positive family history. The Framingham risk score [20] was calculated based on the corresponding variables from the HUNT2 database (age, HDL-cholesterol, total cholesterol, systolic blood pressure, antihypertensive treatment, smoking and diabetes). To classify the metabolic syndrome, a modified set of criteria based on The International Diabetes Federation consensus [21] were used. The criteria were 1) *central obesity*, (men: waist circumference ≥94 cm; women: waist circumference ≥80 cm) plus two of the following four criteria 2a) *low HDL cholesterol* (men <1.03 mmol/L; women <1.29 mmol/L), 2b) *hypertension* (systolic blood pressure ≥130 mmHg or diastolic blood pressure ≥85 mm Hg, or treatment for hypertension), 2c) *fasting plasma glucose* ≥*5.6 mmol/L* or previously diagnosed *type 2 diabetes*, 2d) *fasting triglycerides >1.7 mmol/L*.

### Genotyping

DNA was extracted from peripheral blood leukocytes at the HUNT biobank using a commercial kit (Puregene, Gentra Systems, Minneapolis, MN) or by a robotic method (Autopure LS, Gentra Systems). Genotyping was performed using a combination of polymerase chain reaction (PCR) and pyrosequencing. All of the single nucleotide polymorphisms (SNPs) are found in the online database http://www.ncbi.nlm.nih.gov/snp/. The sequences were based on **ENSG00000165471** (*MBL2*), **ENSG00000085265** (*FCN1*), **ENSG00000160339** (*FCN2*), and **ENSG00000142748** (*FCN3*) in Ensemble release 65.

**Figure 1:**
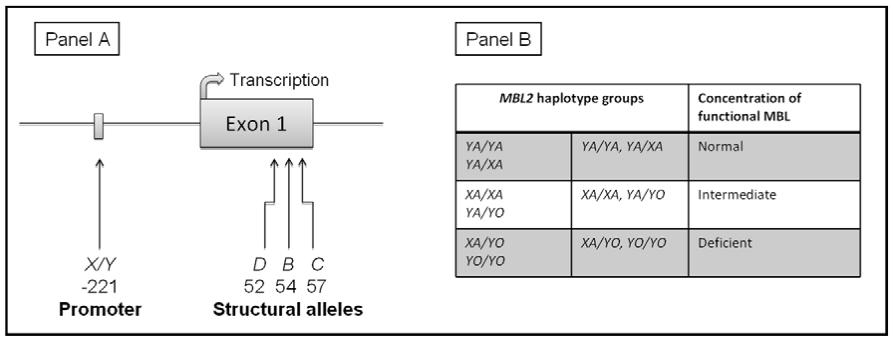
*MBL2* gene and haplotypes. Panel A: Simplified figure of the investigated *MBL2* polymorphisms. Wild type allele is *A.* Panel B: *MBL2* haplotypes and corresponding concentrations of functional MBL.

Four different SNPs in *MBL2* were investigated (Figure 1, Panel A). Three of them are in exon 1 and give rise to the structural alleles *B* (codon 54, rs1800450), *C* (codon 57, rs1800451) and *D* (codon 52, rs5030737). Wild type is denoted *A*. The fourth is considered the most important promoter polymorphism: *X/Y* (rs7096206). These SNPs are inherited in haplotypes. To simplify the interpretation, data are presented by pooling the structural alleles *B–D* to one allele denoted *O* [4]. The structural alleles are always found on a *Y* promoter background, thus we used the term *YO* to denote this defective haplotype. Combining the promoter variant with the *A* and *O* alleles results in 6 haplotypes, as shown in Figure 1, Panel B. These haplotypes were further combined into three haplotype groups: *normal (YA/YA, YA/XA)*, *intermediate (XA/XA, YA/YO)* or *deficient (XA/YO, YO/YO)*, which correspond to serum concentrations of functional MBL [5].

One common polymorphic site in the promoter of the ficolin-1 gene (*FCN1* −542, rs10120023) was genotyped. In the ficolin-2 gene (*FCN2*), two amino acid substituting SNPs in exon 8 were included. They are known to cause increased (*FCN2* +6424, rs7851696) and reduced (*FCN2* +6359, rs17549193) binding capacity of the protein to *N*-acetylglucosamine, respectively [22]. The gene coding for ficolin-3 (*FCN3*) is less polymorphic, but a frame-shift variation in position +1637 (rs28357092) of *FCN3* is known to cause a 50% reduction of serum ficolin-3 in heterozygotes, and total ficolin-3 deficiency in homozygotes [23]. Despite its low allele frequency, this SNP was also included.

Four different PCR reactions were set up: *MBL2* exon 1, *MBL2* promoter, both *FCN2* SNPs, and *FCN1* and *FCN3* in the same reaction. The primers are available as supporting information (Table S1). One primer in each pair was biotinylated. Evaluation of the PCR products by agarose gel electrophoresis showed specific bands of the expected molecular weights. The PCR product was further used for pyrosequencing. We chose the pyrosequencing platform because it has been successfully used for *MBL2* genotyping [24]. Pyrosequencing was performed with a standard protocol on Pyro sequencer PSQ 96MA (Pyrosequencing AB; Biotage, Uppsala, Sweden), using a commercially available kit (PyroMark Gold Q96 Reagents, Qiagen, Germany).

### Statistical analyses

In the pilot study, the Chi-square test was employed to compare the distribution of *MBL2* haplotype groups between patients with and without significant coronary artery stenosis. Logistic regression modelling was used to adjust for the classical cardiovascular risk factors. Plasma MBL concentrations were compared among patients with different *MBL2* haplotypes using the Kruskal-Wallis test.

Based on an expected frequency of 0.08 of the *MBL2* combined low expressing haplotype (*YO/YO*+*XA/YO*) in Caucasians, a power calculation was performed for the case-control study. To detect an odds ratio (OR) of 2.0, assuming a power of 80% and a 5% significance level, 320 persons were required in each group. In order to account for possible variations in the genotype distribution in small datasets, 370 persons were included in each group. The study population was too small for analyses stratified by gender.

McNemar's test was used to compare numbers of discordant pairs. Due to non-normal distribution of several variables, the Wilcoxon signed rank test was used to evaluate differences in continuous and ordinal variables between pairs. The Kruskal-Wallis test was used to compare serum total cholesterol, total cholesterol – HDL cholesterol ratio, and triglycerides within the *MBL2* haplotype groups. The Chi-square test was used for comparison of allele frequencies and of frequencies of participants with hypercholesterolemia within the *MBL2* haplotype groups. Deviation from the Hardy-Weinberg equilibrium was calculated by using the chi-square test.

Conditional logistic regression was performed to evaluate associations between the three inferred *MBL2* haplotype groups and risk of MI. Further models were developed, where traditional risk factors (hypertension, hypercholesterolemia, smoking, diabetes and BMI (continuous)), the Framingham risk score or the metabolic syndrome were also included.

All tests were two-sided and the results are presented as means, ORs or HRs (with 95% confidence intervals (CI)). To avoid false positive conclusions, the alpha level of significance for the comparisons of haplotype frequencies between cases and controls were obtained by permutation testing, using 10,000 permutations. By this method, which is considered the gold standard, a result is significant if the observed p-value is lower than the empirical p-value found under permutation. For other tests, p-values below 0.05 were considered statistically significant. Pilot data was analysed with SPSS for Windows, version 19.0.0 (IBM, New York, USA). Permutation testing was performed using the R package, version 2.14.1 (http://www.r-project.org). All other analyses were performed with Stata/MP for Mac, version 11.2, (Stata Corp., College Station, Texas, USA).

## Results

In the pilot study, male gender, smoking and hypercholesterolemia were more frequent among the patients with significant coronary artery disease [13]. There was an increased frequency of the low *MBL2* haplotype group among patients with significant coronary artery stenosis (p = 0.050, Table 1). The association remained after adjustment for classical risk factors (p = 0.052). As found in previous studies [5], plasma MBL concentrations corresponded closely to the haplotype groups (p<0.0001, Table 1).

**Table 1:** Data on *MBL2* haplotypes and MBL plasma concentrations from the pilot study.

	*MBL2* haplotype			
Coronary artery stenosis	*YA/YA, YA/XA*	*XA/XA, YA/YO*	*XA/YO, YO/YO*	p-value
No (103)	57 (55%)	36 (35%)	10 (10%)	
Yes (131)	75 (56%)	31 (23%)	25 (19%)	0.05
**MBL plasma concentration** (µg/L)	1797 (1618–1976)	373 (289–456)	13 (1–25)	<0.0001

Background characteristics of MI cases and their matched controls are displayed in Table 2. Among cases, baseline measurements of conventional risk factors indicated higher risk of MI in cases than controls: cases had higher BMI, WHR, Framingham risk score and a more unfavourable lipid profile. Furthermore hypertension, diabetes, current smoking, the metabolic syndrome and family history of MI were also more frequent among cases. Creatinine concentrations were similar in the two groups, and were below 140 µmol/L in all participants, indicating no severe chronic renal failure. Mean age at MI was 53 years (range 29–62 years).

**Table 2:** Baseline characteristics.

	Cases	Controls	p-value
	(n = 370)	(n = 370)	
**Gender**, female/male	88/282		---
**Age**, years	48 (47–48)		---
**BMI** *, kg/m^2^	27.4 (27.0–27.8)	26.5 (26.1–26.9)	0.003
**WHR** †	0.89 (0.88–0.90)	0.88 (0.87–0.89)	0.011
**Hypertension**	194 (52%)	162 (44%)	0.015
Systolic blood pressure	140 (139–142)	136 (135–138)	0.002
Diastolic blood pressure	85 (84–86)	83 (82–84)	0.003
**Hypercholesterolemia**	242 (65%)	146 (39%)	<0.0005
**Diabetes mellitus**	13 (4%)	4 (1%)	0.049
**Total cholesterol, mmol/L**	6.8 (6.6–6.9)	6.0 (5.9–6.2)	<0.0005
**Triglycerides, mmol/L**	2.53 (2.35–2.70)	2.05 (1.91–2.18)	<0.0005
**HDL cholesterol, mmol/L**			
Women	1.37 (1.29–1.45)	1.49 (1.40–1.58)	0.024
Men	1.13 (1.08–1.17)	1.22 (1.18–1.26)	<0.0005
**Smoking**			
Never	68 (19%)	114 (32%)	
Former	67 (18%)	81 (23%)	
Current	228 (63%)	156 (44%)	<0.0005
**Framingham risk score**			
Women	13.1 (12.0–14.2)	9.3 (8.1–10.5)	<0.0005
Men	13.4 (13.0–13.9)	11.6 (11.1–12.1)	<0.0005
**Metabolic syndrome**	37 (10%)	20 (5%)	0.022
**Family history** ‡	100 (27%)	54 (15%)	0.001

* Body mass index (BMI).

† Waist hip ratio (WHR).

‡ Myocardial infarction before 60 years in first-degree relatives.

There were no significant deviations from the expected Hardy-Weinberg distributions in the control group (for structural alleles, p = 0.39). Frequencies of *MBL2* haplotypes are given in Table 3. There were higher frequencies of variant haplotypes causing MBL deficiency among cases, compared to controls (p = 0.025, alpha level by permutations = 0.028). Ficolin genotypes are shown in Table 4. There were no significant differences between cases and controls. For *FCN1* −542, however, the number of homozygous individuals was higher in the control group (p = 0.07, recessive model). Frequencies of *MBL2* and ficolin alleles are available as supporting information (Table S2 and Table S3).

**Table 3:** Haplotype frequencies for *MBL2*.

	*MBL2* genotypes		*MBL2* haplotypes		*MBL2* recessive model	
	Cases	Controls	Cases	Controls	Cases	Controls
***YA/YA***	112 (30%)	117 (32%)				
***YA/XA***	86 (23%)	100 (27%)	198 (54%)	217 (59%)		
***XA/XA***	18 (5%)	12 (3%)				
***YA/YO***	91 (25%)	108 (29%)	109 (29%)	120 (32%)	307 (83%)	337 (91%)
***XA/YO***	43(12%)	24 (7%)				
***YO/YO***	20 (5%)	9 (2%)	63 (17%)	33 (9%)	63 (17%)	33 (9%)
**p-value**	**0.025**		**0.005**		**0.001**	
Alpha level by permutation	0.028		0.023		0.029	

**Table 4:** Genotype frequencies for *FCN1*, *FCN2* and *FCN3*.

	Cases	Controls	p-value
	***FCN1*** ** -542 G/A**		
***G/G***	148 (40%)	137 (37%)	
***G/A***	177 (48%)	170 (46%)	
***A/A***	45 (12%)	63 (17%)	0.19*
	***FCN2*** ** +6359 C/T**		
***C/C***	181 (49%)	196 (53%)	
***C/T***	157 (42%)	141 (38%)	
***T/T***	32 (9%)	33 (9%)	0.46
	***FCN2*** ** +6424 G/T**		
***G/G***	289 (78%)	295 (80%)	
***G/T***	77 (21%)	71 (19%)	
***T/T***	4 (1%)	4 (1%)	0.86
	***FCN3*** ** +1637 C/−** †		
***C/C***	363 (99%)	364 (99%)	
***C/−***	5 (1%)	4 (1%)	
***−/−***	0 (0%)	0 (0%)	0.74

* *FCN1* recessive model: p = 0.069.

† 4 missing.

Conditional logistic regression showed that variant *MBL2* haplotypes causing MBL deficiency were positively associated with MI (Table 5). The three haplotype groups, corresponding to functional MBL concentration, were used in the analyses. The odds for MBL deficiency among MI cases was twice as high as in controls (OR = 2.04, 95%CI 1.29–3.24, p = 0.003), and adjustment for conventional cardiovascular risk factors (hypertension, BMI, hypercholesterolemia, diabetes, and smoking, OR 2.02, 95%CI (1.17–3.47), p = 0.012), the Framingham risk score, or presence/absence of the metabolic syndrome did not substantially influence the association (Table 5). There was missing data on family history in 131 case-control pairs (71 cases and 66 controls, p = 0.64), and information on family history was therefore not included in the analysis. There were no associations between lipid profile and *MBL2* haplotypes (total cholesterol: cases p = 0.66, controls p = 0.43; total cholesterol – HDL cholesterol ratio: cases p = 0.68, controls p = 0.14; triglycerides: cases p = 0.69, controls p = 0.16; frequency of hypercholesterolemia: cases p = 0.28, controls p = 0.60).

**Table 5:** Conditional logistic regression analyses, *MBL2* functional groups.

	OR	95% CI	p-value
*Model 1*			
***YA/YA, YA/XA***	1		
***XA/XA, YA/YO***	1.01	(0.72–1.41)	0.96
***XA/YO, YO/YO***	2.04	(1.29–3.24)	0.003
*Model 2* *			
***YA/YA, YA/XA***	1		
***XA/XA, YA/YO***	1.02	(0.73–1.44)	0.89
***XA/YO, YO/YO***	1.91	(1.19–3.08)	0.008
*Model 2 – Adjusted for classical risk factors* * †			
***YA/YA, YA/XA***	1		
***XA/XA, YA/YO***	1.26	(0.84–1.88)	0.27
***XA/YO, YO/YO***	2.02	(1.17–3.47)	0.012
*Model 2 – Adjusted for Framingham risk score* * ‡			
***YA/YA, YA/XA***	1		
***XA/XA, YA/YO***	1.19	(0.80–1.77)	0.39
***XA/YO, YO/YO***	2.09	(1.22–3.59)	0.007
*Model 3 – Adjusted for metabolic syndrome* §			
***YA/YA, YA/XA***	1		
***XA/XA, YA/YO***	1.06	(0.76–1.49)	0.73
***XA/YO, YO/YO***	1.98	(1.25–3.16)	0.004

* 26 pairs excluded because one or more missing values.

† Adjusted for classical risk factors: Hypertension (BP>140/90 or current use of antihypertensive medication), body mass index (kg/m^2^, continuous), hypercholesterolemia (total cholesterol >6.2 mmol/L), diabetes (yes/no) and smoking (never/former/current).

‡ Adjusted for Framingham risk score (age, HDL-cholesterol, total cholesterol, systolic blood pressure, smoking and diabetes).

§ 3 pairs excluded because one or more missing values.

## Discussion

In this population-based case-control study we found that variant *MBL2* haplotypes causing MBL deficiency were associated with a doubling of the risk of MI at middle age (before the age of 62 years). The association was independent of conventional risk factors for MI. Based on the pilot study where associations among variant *MBL2* haplotypes, low plasma concentrations of MBL, and increased frequency of significant coronary artery stenosis were demonstrated, increased atherosclerosis is a probable explanation of this finding.

Previous studies support our finding. However, those studies were performed among patients with severe atherosclerosis [6] or other predisposing conditions, such as a high prevalence of coronary artery disease [7] or inflammatory diseases, i.e. systemic lupus erythematosus [11], rheumatoid arthritis [25] or type 2 diabetes mellitus. Our results also suggest that MBL deficiency is a particularly strong risk factor for cardiovascular events among young to middle-aged and apparently healthy individuals.

Although the study population was relatively young, conventional cardiovascular risk factors, including hypertension, hypercholesterolemia, smoking and high BMI were also associated with increased risk of MI. Despite incomplete data, there was also a positive association of family history of MI with MI risk. We chose to study people at middle age, anticipating that underlying causes of an early MI would be more likely to be genetic compared to an older age, when non-genetic causes may dominate. Another reason was our previous finding that *MBL2* was more strongly associated with severe atherosclerosis in the youngest patients going through coronary surgery [6]. At older age, the importance of genetic factors may be difficult to distinguish from the impact of environmental and life style factors and comorbidities.

Previous studies support a cardio-protective role of MBL [26] and activation of the lectin pathway. Rats with MBL deficient macrophages fed on a high-cholesterol diet were more likely to develop atherosclerotic lesions, which may be explained by reduced removal of apoptotic cells and debris by MBL [12]. Remaining apoptotic cells may then increase inflammation and lead to formation of vulnerable plaques, which can result in MI, independent of the extent of atherosclerotic disease. In humans, variant *MBL2* alleles may be correlated with increased carotid plaque area [9] and MBL deficient individuals may also have higher postprandial lipid values [27], which in turn may contribute to the development of atherosclerosis [28]. In our study, we did not observe any association between lipid values and *MBL2* haplotypes. Although the link between infections and atherosclerosis is not verified, a combination of MBL deficiency and infection was related both to the development of coronary artery disease [8] and to reduced flow-mediated vasodilation [29], which is an early marker of endothelial dysfunction. Those results imply plausible mechanisms that may contribute to an increased risk of atherosclerosis in the presence of MBL deficiency.

On the other hand, others have found that high serum MBL-concentrations [10] and wild type *MBL2* may be associated with increased risk of cardiovascular disease. A dual effect of MBL has been suggested, as both high and low serum concentrations of MBL were correlated with increased intima-media thickness of the carotid artery in persons with rheumatoid arthritis [25]. Speidl et al have suggested that activation of the complement cascade by the alternative pathway may be proatherogenic as a result of inflammation, whereas activation through the lectin and classical pathways may have protective effects [30]. Activation of the lectin pathway of complement through MBL and MBL-associated serine proteases may activate the coagulation cascade and lead to thrombosis [31] which could increase the risk of MI. Furthermore, it should be noted that MBL and activation of the lectin pathway appears to be central in ischemic reperfusion injury, which may blur the relative cardioprotective effects of MBL in atherosclerosis [32], [33]. Thus, current evidence indicates that MBL has several effects that either may increase or reduce the risk of cardiovascular events, and the clinical importance in different settings is not fully understood

Analysis of haplotypes with known functional consequences and ensuring stringent alpha levels of significance by permutation testing strengthen the probability of a causal relationship, even if the design of our study did not allow direct causal inference. Our results corroborate that genetically determined MBL deficiency is linked to atherosclerosis. However, we cannot exclude that high MBL concentration and an “eager” complement system may also be harmful in the atherosclerotic process under some circumstances.

None of the ficolin polymorphisms were significantly related to MI. One may speculate that being homozygous for *FCN1 −542* yields some protection, but little is known about the effects of this genetic variation, and more research is needed.

This study was not designed to test improvement of risk prediction. However, the results may generate new hypotheses regarding pathophysiology.

### Study Limitations

There are some limitations to our study. *MBL2* haplotypes were not assessed in all MI patients from the HUNT2 study; hence it is not known whether the associations are present in older individuals. Serum was not available at the time of the genotyping. However, our pilot study and other previous studies have shown that serum concentrations of functional MBL correspond closely to the genotypes [5]. The risk for an early MI is also influenced by other genetic factors, which were not included in the present study. Non-linearity in the light response following incorporation of more than five nucleotides may play a role in the assessment of homopolymeric regions like the one in *FCN3*. All samples with uncertain results were therefore re-sequenced manually. The results were not replicated in a similar cohort, as they were partly confirmatory. Because the included polymorphisms are located closely together and are not covered in previously published genome-wide association studies on early MI, it was not possible to use such data for replication. Although the population in Norway is assumed to be generally representative for the Caucasian population, we cannot exclude the possibility that MBL may be more important in relation to cardiovascular disease in this population compared to others.

### Conclusions

The *MBL2* haplotypes corresponding to functional MBL deficiency were associated with a doubling of the risk for MI in individuals younger than 62 years of age, independent of conventional risk factors. The findings confirm our hypothesis and support that MBL deficiency may lead to increased atherosclerosis or development of vulnerable plaques.

## Supporting Information

Table S1 **Primers.**
(DOCX)

Table S2 **Distribution of** ***MBL2*** **alleles.**
(DOCX)

Table S3 **Allele frequencies for** ***FCN1*** **,** ***FCN2*** **and** ***FCN3*** **.**
(DOCX)
