# Supplementary material for: Mannose-Binding Lectin Deficiency Is Associated with Myocardial Infarction: The HUNT2 Study in Norway
Source: PLoS One. 2012 Jul 27;7(7):e42113. doi: 10.1371/journal.pone.0042113 (PMC3407165; doi:10.1371/journal.pone.0042113)
Supplement: Table S1 — Primers. (DOCX) [file pone.0042113.s001.docx]

Table S1. Primers

| **Gene** | **Primer** | **Primer sequence (5´-3´)** |
| --- | --- | --- |
| *MBL2* exon 1, codon 52, 54 and 57 (*D*, *B*, *C*) | | |
|  | Forward | CCTTCCCTGAGTTTTCTCAC |
|  | Reverse | AACAGCCCAACACGTACCTG |
|  | Sequencing | CGTACCTGGTTCCCCCTTTTCT |
| *MBL2* promoter -221 (*X/Y*) | | |
|  | Forward | TGGTGTGAGAAAACTCAGGGAAG |
|  | Reverse | GCACGGTCCCATTTGTTCTC |
|  | Sequencing | CTGGAAGACTATAAACATGCTT |
| *FCN1* -542 | | |
|  | Forward | TCCCAAATACTATTTCCATCATATC |
|  | Reverse | CTTCAATTTCTCCAGCTGTAACT |
|  | Sequencing | ATCTTGCACCAGCCC |
| *FCN2* +6359, +6424 | | |
|  | Forward | TCACATTTCCTCCTGCACAGG |
|  | Reverse | TTGACACATGGCAGTTTTTGTAC |
|  | Sequencing +6359 | CACAGGAGATTCCCTGA |
|  | Sequencing +6424 | GATCTTAACACCGGAAATT |
| *FCN3* +1637 | | |
|  | Forward | GAGCCAGGGCGCCACCTT |
|  | Reverse | CCCCCCTCGGTGTCCATGT |
|  | Sequencing | CTACCTGAGGGCAGG |
