# Supplementary material for: Mannose-Binding Lectin Deficiency Is Associated with Myocardial Infarction: The HUNT2 Study in Norway
Source: PLoS One. 2012 Jul 27;7(7):e42113. doi: 10.1371/journal.pone.0042113 (PMC3407165; doi:10.1371/journal.pone.0042113)
Supplement: Table S2 — Distribution of MBL2 alleles. (DOCX) [file pone.0042113.s002.docx]

Table S2. Distribution of *MBL2* alleles

|  | **Cases** | **Controls** |
| --- | --- | --- |
| **SUM *A/A*** | **216 (58%)** | **229 (62%)** |
| *A/B* | 82 (22%) | 79 (21%) |
| *A/C* | 9 (2%) | 4 (1%) |
| *A/D* | 43 (12%) | 49 (13%) |
| **SUM *A/O*** | **134 (36%)** | **132 (36%)** |
| *B/B* | 7 (2 %) | 2 (0.5%) |
| *B/C* | 2 (0.5%) | 0 (0%) |
| *B/D* | 7 (2 %) | 4 (1 %) |
| *C/C* | 0 (0%) | 0 (0%) |
| *C/D* | 0 (0%) | 1 (0.3%) |
| *D/D* | 4 (1 %) | 2 (0.5%) |
| **SUM *O/O*** | **20 (5.4%)** | **9 (2.4%)** |
| *Allele frequency A* | 566 / 740 (76%) | 590 / 740 (80%) |
| *Allele frequency O* | 174 / 740 (24%) | 150 / 740 (20%) |
| *Y/Y* | 223 (60%) | 234 (63%) |
| *X/Y* | 129 (35%) | 124 (34%) |
| *X/X* | 18 (5%) | 12 (3%) |
| *Allele frequency Y* | 575 / 740 (78%) | 592 / 740 (80%) |
| *Allele frequency X* | 165 / 740 (22%) | 148 / 740 (20%) |
