# Supplementary material for: Mannose-Binding Lectin Deficiency Is Associated with Myocardial Infarction: The HUNT2 Study in Norway
Source: PLoS One. 2012 Jul 27;7(7):e42113. doi: 10.1371/journal.pone.0042113 (PMC3407165; doi:10.1371/journal.pone.0042113)
Supplement: Table S3 — Allele frequencies for FCN1 , FCN2 and FCN3 . (DOCX) [file pone.0042113.s003.docx]

|  | **Cases** | **Controls** | **p-value** |
| --- | --- | --- | --- |
| ***FCN1 -542*** | | | |
| ***G*** | 473/740 (64%) | 444/740 (60%) |  |
| ***A*** | 267/740 (36%) | 296/740 (40%) | 0.12 |
| ***FCN2 +6359*** | | | |
| ***C*** | 519/740 (70%) | 533/740 (72%) |  |
| ***T*** | 221/740 (30%) | 207/740 (28%) | 0.42 |
| ***FCN 2 +6424*** | | | |
| ***G*** | 655/740 (89%) | 661/740 (89%) |  |
| ***T*** | 85/740 (11%) | 79/740 (11%) | 0.62 |
| ***FCN3 +1638*** | | | |
| ***C*** | 731/740 (99%) | 732/740 (99%) |  |
| ***-*** | 5/740 (0.7%) | 4/740 (0.5%) | 0.74 |

Table S3. Allele frequencies for *FCN1*, *FCN2* and *FCN3*
